# Developing and testing the ExerciseGuide UK website for people with lung cancer: reflections on the added value of patient and public involvement within a doctoral degree

**DOI:** 10.1186/s40900-022-00395-1

**Published:** 2022-11-30

**Authors:** Jordan Curry, Helen Roberts, Alan Smith, Diane Riley, Mark Pearson, Cynthia C. Forbes

**Affiliations:** 1 Wolfson Palliative Care Research Centre, Hull York Medical School, University of Hull, Kingston Upon Hull, Cottingham Road, Hull, HU6 7RX UK; 2 Hull York Medical School, University of Hull, Kingston Upon Hull, Cottingham Road, Hull, HU6 7RX UK; 3 Involve Hull, Hull York Medical School, University of Hull, Kingston Upon Hull, Cottingham Road, Hull, HU6 7RX UK

**Keywords:** Patient and public involvement, Digital health, eHealth, Usability, Co-design, Lung cancer, Physical activity

## Abstract

**Background:**

Lung cancer has one of the highest incidence and mortality rates worldwide. Physical activity can provide those diagnosed with lung cancer with several physical and psychological benefits. However, the examination of digitally delivered physical activity to those with lung cancer is not as researched as other common cancers. Often, those diagnosed with lung cancer are older adults (65 years or older). Older adults are often wrongly assumed to lack digital skills, interest, and not engage with digital technology regularly. Although individuals are interested, would involving older people in designing of websites and apps result in better engagement?

**Main body:**

In this article, the authors discuss the process of adapting a digital platform with a patient and public involvement group to provide those who have received a lung cancer diagnosis with a tailored physical activity program and health educational modules. We discuss the influence of recurrent patient and public involvement on the study, the patient and public involvement members, and the doctoral researcher.

**Conclusion:**

Working with a patient and public involvement group over several months, especially potential users of a digital intervention, may enhance its relevance, accessibility, and usability. By engaging with patients, family, or caregivers for someone with lung cancer, the doctoral student gained insight into the needs of the study population and what to consider during development. All group members expressed their interest and enjoyment in their involvement, and several are now active members of a wider patient and public involvement network.

## Plain English summary

This commentary describes how patient public involvement has been used to adapt a website called ExerciseGuide UK. This website provides a personalised physical activity program and education to those diagnosed with lung cancer. The programme is altered to allow for each patient’s capabilities. Reflections on how the study affected both the researcher and the PPI members are discussed. The commentary gives the patient and public involvement members a voice in their involvement experience. It highlights the difference that their sustained involvement made to the study, the doctoral researcher, and those who were involved. Globally, lung cancer is a leading cause of cancer-related death and remains one of the most common types of cancer. Digital technology, such as websites, mobile applications, and smart wearable devices (e.g., Apple Watch and Fitbits), have increasingly been used in health research over the last few decades. Since the beginning of the COVID-19 pandemic in 2020, research into digital technology has increased rapidly. Individuals diagnosed with lung cancer may experience a large number of physically and emotionally limiting symptoms, such as a higher risk of severe illness due to infections. With the large symptom burden they experience, digital technology may provide alternative and more accessible methods which can be altered to suit and help their specific needs. The patient and public involvement group members had all either been diagnosed with lung cancer, cared for someone with lung cancer, or experienced lung cancer in their family.

## Background

**Fig. 1:**
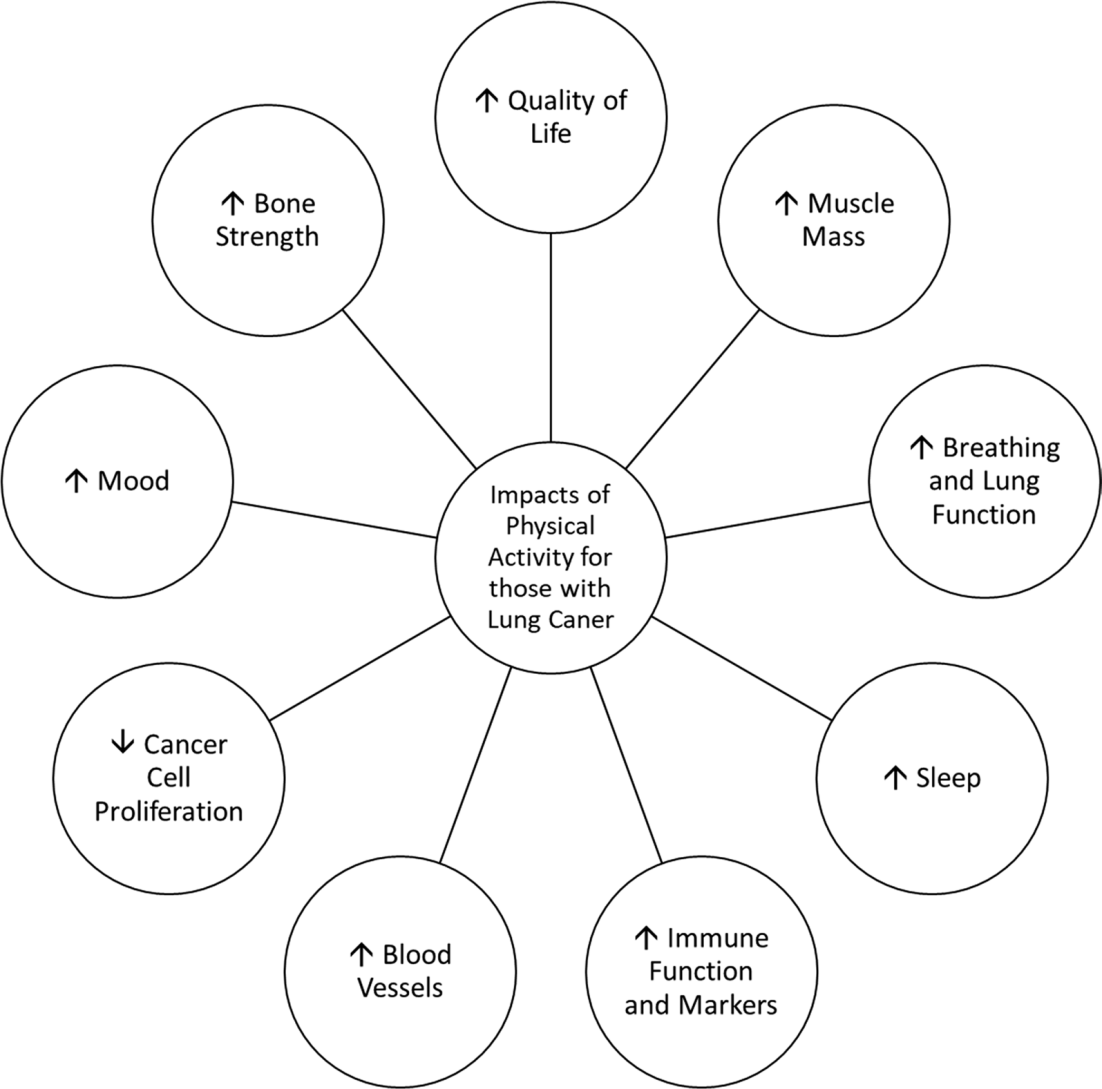
Summary of the positive impacts physical activity can have for those living with and beyond lung cancer

Lung cancer is the leading cause of cancer-related deaths globally [1]. Patients diagnosed with lung cancer often have a higher burden of symptoms than patients with other prevalent cancers and are less likely to receive support to manage these symptoms [2, 3]. Physical activity has many benefits for the physical and mental health of those diagnosed with lung cancer (see Fig. 1).

Digital technology has been an emerging field of health research in recent decades. Digital technology has demonstrated that it can provide tailored physical activity programs to people at relatively low cost and is generally easily accessible [4]. However, patients do not use such tools as often as they could, despite expressing interest [4]. Therefore, the question remains, why are users not engaging with digital health tools? One common thought is that such tools lack user perspectives and input during the design phase [5, 6].

This study involved adapting an existing exercise website (ExerciseGuide) to provide a personalised physical activity program and education guided by behaviour change techniques that aim to overcome possible activity barriers for those who have received a lung cancer diagnosis.

This commentary focuses on how giving patient and public members a voice in this research was important, the influence of the patient and public involvement (PPI) group on the study, how involvement in this doctoral PPI impacted the PPI group, and how the doctoral researcher worked with the group online to adapt and co-design the website (ExerciseGuide UK) to test in a feasibility study.

## Main body

### Doctoral research and ExerciseGuide UK

The doctoral research concerning this commentary focuses on exploring ExerciseGuide UK, a web-based platform that allows tailored information for those who have received a lung cancer diagnosis based on IF–THEN rules. Essentially, *IF* an individual selects a statement, *THEN* they will be shown a pre-determined answer. ExerciseGuide UK has been adapted from previous versions of ExerciseGuide [7–11]. A detailed protocol of the adaptation and redevelopment of ExerciseGuide UK has been published [12]. ExerciseGuide UK is still hosted online but is currently closed for new user enrolment (https://www.exerciseguide.org.uk). The PPI was a key component in ExeriseGuideUK's adaptation and new content development.

### Purpose of patient and public involvement

The purpose of conducting sustained PPI was to ensure ExerciseGuide UK was adapted and new content developed appropriately for those with a lung cancer diagnosis. By sustained PPI, we mean that we worked with our PPI group over a period of several months. ExerciseGuide UK was collaboratively revised through sustained PPI and feedback from qualitative Think-Aloud interviews. The number of PPI members involved varied over the three workshops. Four members attended the initial workshop. Three attended workshop two (two of the original group died in the intervening period, and one new person joined the group), and three attended workshop three. In total, five people were involved. Think-Aloud interviews were conducted with seven individuals who had a lung cancer diagnosis. There was no cross-over between the PPI members and the Think-Aloud participants. Participants performed several pre-determined tasks on the website via Zoom and were encouraged to actively speak their actions and thoughts aloud. Further information regarding the Think-Aloud reviews are discussed in the published protocol [12]. All thoughts identifying areas of concern were brought to the PPI group for potential change.

### Workshop delivery and feedback

In this section, we will provide a summary of the process taken to conduct the PPI workshops. Further information regarding key methods is presented in the published protocol paper [12].

#### Workshops

A series of face-to-face workshops were initially planned with support and guidance from the research team, who had previous experience and expertise in PPI. The first two workshops aimed to review the content of the ExerciseGuide UK program and explore four key areas:

1. Understanding of physical activity concerning lung cancer
2. Barriers to engaging in physical activity and digital technology
3. Creating clear materials for study participants
4. Module development within ExerciseGuide UK

The third workshop reviewed the findings from Think-Aloud interviews conducted among people with lung cancer. One final workshop was conducted to reflect on how the PPI shaped the intervention and the study. A recruitment flyer was developed and disseminated online (via Twitter) and through support groups for those affected by a diagnosis of lung cancer. Three members joined the PPI group in response to the recruitment flyer. Two members were invited from an existing PPI network, Involve Hull, based on their personal experiences of lung cancer.

Due to the Coronavirus-19 pandemic, all PPI workshops were adapted for online delivery. The research team were cautious about this transition to solely virtual PPI and lacked experience in this way of working. In practice, this increased the geographical reach of the PPI by involving people from outside the local area, including people living in cities and towns in the southwest and northwest of England. One member highlighted the positive benefits of virtual PPI in terms of how they felt able to engage with each other:

The medium of Zoom has certainly enabled me to participate living far away – PPI Member 2

I am more prepared to break in and make my own contribution in this [Zoom], particularly online, instead of in a room where I would need to attract your attention, maybe stand up, people looking at me… it's much less intimidating – PPI Member 2

However, although the geographical reach increased, individuals lacking digital skills or with limited or no access to digital technology were not included; therefore, their insights were not captured.

#### Feeding back to the group

All PPI sessions were recorded with group consent. Following the first two PPI workshops, a summary of the discussion was shared with the group. A traffic light colour coding method was used to identify which ideas would be taken forward, which were under consideration, and which could not be addressed within this study. The feedback allowed the group to see that their input had been heard and understood, provided further opportunities for reflection and ideas, and ensured the researchers recorded their comments accurately. The summaries produced were well received by the PPI group and encouraged them to sustain their engagement:

It is certainly motivation for me to get all this feedback and how it has been taken on board. Sometimes is it very difficult to know whether your input has been of any value or any notice of, so no, I really appreciate that, and it will certainly make me come back and do future PPI roles – PPI Member 1

I think also you get more people who are keen to contribute when they can see their contribution has been seen, recognised, and been acted on…people won't actually contribute change if they don't feel that change is being acted on – PPI Member 2

### Reflections of patient and public involvement

#### Study revisions

After analysing seven Think-Aloud interviews, 24 proposed revisions to the website were brought to the PPI group for discussion. There were five main themes: (1) Understanding and Clarity, (2) More Information Needed, (3) Visual, (4) Functionality, and (5) Preferential. The PPI group agreed with 46% of the revisions proposed and, in collaboration with the doctoral researcher, found ways of improving the rest. Most of the proposed revisions centred on ‘More information, ‘Understanding and Clarity’ and ‘Preference’. This process resulted in a consensus on how best to adapt ExerciseGuide UK. Involving the PPI group ensured the right level of information was provided, and that information was clear, suitable, and appropriate for those with a lung cancer diagnosis.

**Fig. 2:**
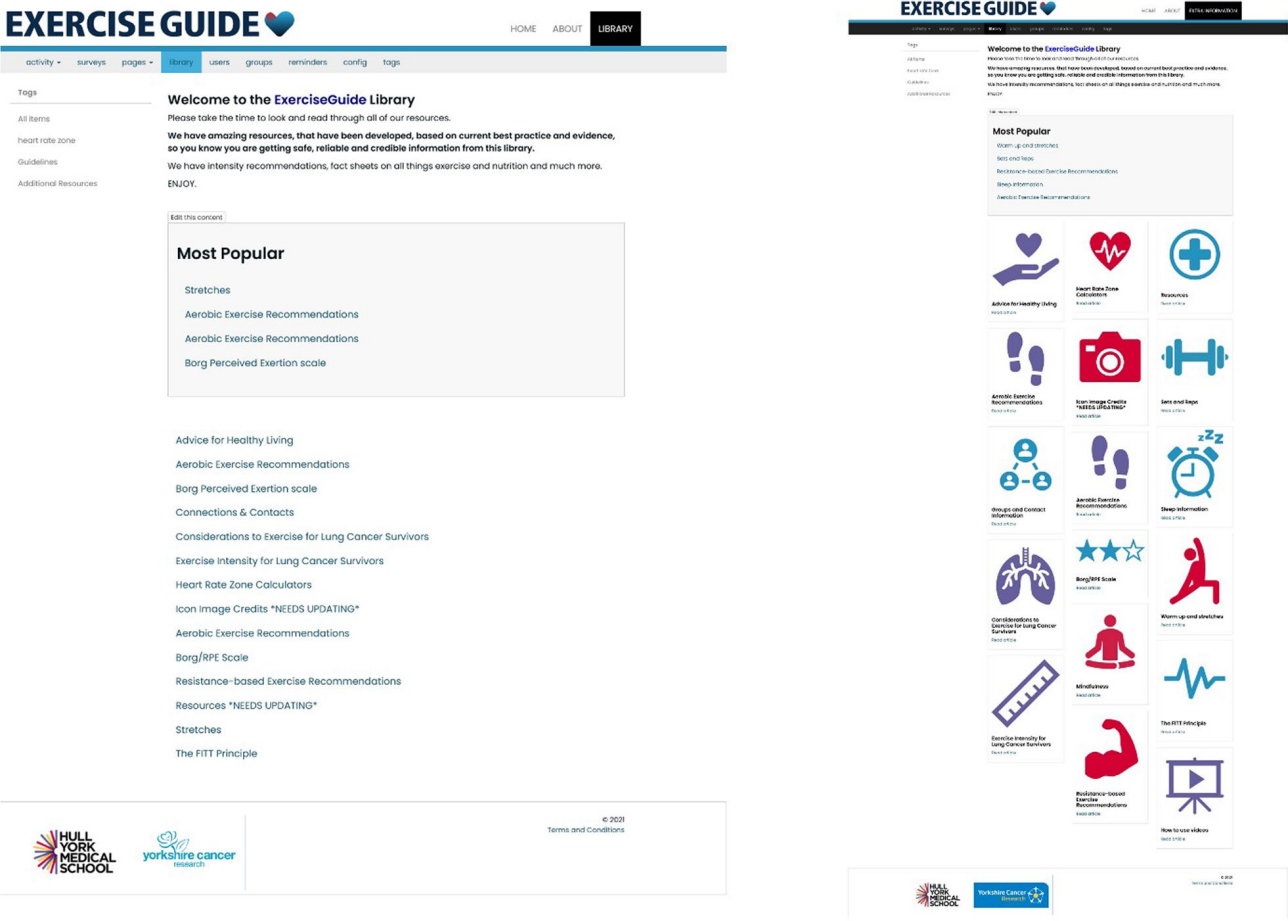
Original (left) and revised (right) screenshots of the Extra Information page (formerly known as Library) on ExerciseGuide UK. Figured taken from [12]

For instance, the landing page of ExerciseGuide UK originally stated the website was for those “living with and beyond lung cancer”. The PPI group did not feel this phrase was clear. Therefore, the statement was revised to read, *This personally tailored website is for those who have received a lung cancer diagnosis and aims to help you to become more active in a fun and educational way!*. Another example was the redesign and renaming of the library of additional resources. The name, Library, was unpopular with those who took part in the qualitative interviews, and the PPI group decided ‘Extra Information’ was more understandable. Figure 2 demonstrates how the design of this page was revised after discussion with the PPI group, with colourful icon thumbnails making the content more visible and navigation much easier.

#### Reflections of the PPI group members

Members of the PPI group commented on the impact that involvement has had on their understanding of PPI in health research.

For me, Involve [Involve Hull] has just opened my eyes up to a whole world that I didn't realise was out there [research and PPI]…it has opened my eyes up so much. To feel you can make a contribution is very energising – PPI Member 1

It has been very interesting to see how much goes into these sorts of PPI activities. I had no idea it was governed by so many rules, regulations, protocols and things – PPI Member 2

Two members commented that their involvement in this PPI positively impacted their perceptions of lung cancer.

It benefits me in realising I was not the only person with this [lung cancer], which you often feel like you are. So, it was interesting to meet others and hear their experiences – PPI Member 2

For me, the good feeling about the fact that you can be involved in it, but for me, personally, like [PPI member 2] said… y'know realising some people with lung cancer can have a good life with it, some quality of life, sometimes they can enjoy together, which we didn't have. – PPI Member 3

#### Doctoral student reflections

From the beginning of this doctoral degree, the primary aim was to adapt and develop an online platform to provide a tailored physical activity program and education. Before redeveloping ExerciseGuide UK, a systematic review was conducted to explore the evidence regarding online supportive care for those diagnosed with lung cancer [13]. The platform underwent initial redevelopment using the findings from the review and existing knowledge gained from prior experience working as an exercise professional. Then, the PPI group was formed to gain feedback on the platform's prototype and assistance with further adaptations. Although the initial prototype was well received, suggestions were made to change certain aspects of ExerciseGuide UK. Reflecting on the process, it would have been beneficial to set up a PPI group before the redevelopment began so that the lived experience could guide the whole process. This way, time would be saved by collaboratively deciding what content and features to develop.

Opening up the website to critique from the PPI group was challenging as a doctoral student. After months of reviewing literature regarding the needs of individuals diagnosed with lung cancer, it was difficult to hear that the platform did not currently meet the needs of those with lung cancer as intended. Doctoral students seek feedback from their supervisors and other senior academics. However, as public involvement at doctoral level is unusual, they are less used to having their work critiqued by public members. Being exposed to feedback from those with lived experience who represent the population an intervention is designed for is a different learning process. It involves learning to accept that knowledge based on personal experience of health and care is equally valid to professional or academic knowledge and its benefits. In this case, the benefits were a better, more relevant and user-friendly website, and the personal learning and development of the doctoral student.

The doctoral student brought the findings from the Think-Aloud interviews to the PPI group for discussion and agreement. Each issue was presented, along with a proposed solution, and the group worked through these to agree how the website would be revised. All PPI and staff members agreed with the accepted changes. This meant that the final prototype was more appropriate for its target population and had greater clarity and overall usability.

## Discussion

Involving the PPI group in the study multiple times clearly improved the final product. Following the Think-Aloud interviews, 54% of the proposed revisions to increase the platform's usability and acceptability were further revised with the PPI group. Even though it was sometimes difficult to hear criticism for a lot of hard work, usability and acceptability issues were likely to remain without this co-design process. Research waste is a serious concern, particularly in health sciences, with up to 85% of health research potentially wasted, possibly due to poor study design and conduct [14]. Ensuring PPI is involved in all research, including doctoral research, may reduce poor design, increase the level of appropriateness and usability for special populations (e.g. older adults), and encourage patient recruitment and retention to allow completion.

Although digital technology was the primary method of PPI during the Coronavirus-19 pandemic, it should not replace face-to-face involvement. Approaching PPI virtually without offering alternative ways of getting involved will exclude those not engaging with digital technology. The authors acknowledge that these individuals may have been excluded.

All members are now active in the University of Hull's PPI network (Involve Hull) and contribute to the university's ongoing research. The PPI contributors involved said being involved in this doctoral PPI was beneficial to them personally. For example, one PPI member highlighted hearing positive stories of others having a diagnosis of lung cancer was valuable to them. Their participation has also been beneficial to their awareness of different lung cancer outcomes, and when their suggestions were visibly heard and implemented, they felt good and motivated. Two PPI members of the PPI group are co-authors (Smith and Riley) of this commentary.

Doctoral students can benefit greatly from working iteratively to develop interventions with the input of PPI. Working closely with the PPI group on designing, adapting, and developing content for the intervention was extremely valuable in ensuring intervention success. Another benefit was a process of in-depth learning about how to address barriers and ease concerns about physical activity and digital technology.

## Conclusion

Although PPI is not mandatory within doctoral research, it can ensure an appropriate, relevant, and acceptable intervention, reduce the possibility of research waste, and provide the research team with knowledge and expertise that they would not otherwise be able to access.

## Data Availability

N/A.

## Abbreviation

PPI: Patient and public involvement
